# Gender-Based Violence During COVID-19 Pandemic: A Mini-Review

**DOI:** 10.3389/fgwh.2020.00004

**Published:** 2020-09-08

**Authors:** Shalini Mittal, Tushar Singh

**Affiliations:** ^1^Amity Institute of Behavioural and Allied Sciences, Amity University, Lucknow, India; ^2^Department of Psychology, Banaras Hindu University, Varanasi, India

**Keywords:** quarantine, gender-based violence, pandemic, domestic violence, COVID 19

## Abstract

**Purpose:** Quarantine is necessary to reduce the community spread of the Coronavirus disease, but it also has serious psychological and socially disruptive consequences. This is known as the quarantine paradox that also includes a surge in the cases of gender-based violence. However, there exists a clear gap of rigorous literature exploring the issue. Hence, the current paper attempts to understand gender-based violence as an aspect of the COVID-19 lockdown. It reviews the pattern of rise in gender violence cases and the resultant psychological and social issues and attempts to create awareness by initiating a discourse urging for change in the response towards the victims of gender-based violence. The paper further attempts to suggest measures to mitigate the issues arising out of gender violence during quarantine.

**Method:** The current paper reviews the literature on the rise of gender-based violence in the times of current and past pandemics. The paper also reviews the published reports in scientific as well as mass media literatures focusing on the rise of gender-based violence during the imposed lockdown, its consequences, and the measures taken by the governments to tackle the issue.

**Results:** The present review reveals that similar to the previous pandemics and epidemics, there has been an alarming rise in the incidents of gender-based violence during the COVID-19 pandemic. The present review further reveals various other risk factors that have been found attributive to the surge of gender-based violence such as economic insecurity and alcohol consumption. The results of the review indicate that despite its global prevalence, gender-based violence has been one of the most neglected outcomes of pandemics. Moreover, the legislatures and services available for such victims are often inadequate and, thus, worsening their situation.

**Conclusion:** Pandemic situations have been found to be associated with advancements in the medical field. However, a part and parcel of this situation is the age-old practice of quarantine that has several negative outcomes. This also includes a surge in gender-based violence that raises serious concerns about the safety of women. As the legislatures provided and measures taken by the governments are falling short in dealing with the issue, a number of non-government organizations are stepping up to provide necessary services to these victims.

## Pandemic and Gender-Based Violence

Quarantine has been an effective measure of controlling infection since the 14th century. The medieval societies were able to establish a link between the emergence of symptoms and the duration of time. The origin of the term is rooted in the health practice related to plague back in 1377 AD when ships were isolated for 30 days and land travelers for 40 days in the sea port of Ragusa (1). However, the earliest record of quarantine can be traced back to 532 AD (2). Since then, the practice of quarantine has been utilized to reduce the spread of contagious diseases. With the declaration of COVID-19 as a global pandemic, there is a mounting pressure on the governments to take measures to reduce the community spread of the disease. Hence, in the absence of a vaccine or effective treatment, going into quarantine for varying periods of time is being adopted as an option by most countries. This has led to a drastic alteration in the day-to-day lifestyle of the individuals. Most of the work is being done from home, and efforts are being made to maintain social distance. These measures are crucial to the protection of healthcare systems. However, just like one coin has two sides, the positive efforts to tackle COVID-19 have negative consequences associated with them. These negative consequences include the risk of losing jobs, economic vulnerabilities, and psychological health issues resulting from isolation, loneliness, and uncertainty, among others. This can be regarded as the quarantine paradox. History has witnessed the weakening of the states in the face of pandemics and outbreaks. The Antonine plague of 161 AD had economically weakened the Roman Empire (3). The Byzantine empire too had suffered weakening of the economic infrastructure during the Justinian plague (4). Past researches indicate that the risk of serious psychological consequences increases with the increase in the duration of the quarantine (5). According to Hawryluck et al. (6) and Reynolds et al. (7), a longer duration of quarantine was found to be associated with increased symptoms of PTSD. Lee et al. (8) reported that the risk of developing PTSD symptoms persisted despite home quarantine. Another downside of quarantine is the increase in cases of gender-based violence that is frequently ignored (9). Gender-based violence is a form of violence targeting a person based on the gender of an individual. It is a complex phenomenon that includes combinations of sexual, physical, and emotional violence and neglect or deprivation (10). CEDAW (Committee on Elimination of Discrimination Against Women) has defined gender-based violence as a form of violence that disproportionately affects women. Some common forms of gender-based violence include sexual violence, violence against women, domestic violence, and harmful traditional practices, such as female genital mutilation. For the present paper, the term gender-based violence has been used to denote different aspects of domestic violence against women.

According to an article published in a national newspaper of India, The Hindu, the National Commission for Women (NCW) recorded a twofold rise in the cases of gender violence (11). Several researches indicate a rise in family violence and sexual violence during and after any large crisis or disaster [e.g., (12, 13)].

## Relation Between Gender-Based Violence and Crisis Situations

Violence has generally been found to increase in the face of pandemics. For instance, Rose (14) reported an erosion of social norms and increase in violence in Bologna, Italy, in the context of plague and natural disaster. According to UNFPA (15), pandemics often lead to breakdowns of social infrastructures thus compounding the already existing weaknesses and conflicts. As a result, the existing gender inequality is worsened by the pandemic situations. It also increases the exposure of children and women to harassment and sexual violence when they try to procure necessities such as water, food, and firewood. Several researches report that gender-based violence is more prevalent in HIV hyper-endemic countries [e.g., (10, 16)]. Researchers have observed a link between the prevalence of HIV epidemic and gender-based violence in India as well (17, 18). A report about rapid gender analysis on COVID-19 by CARE and International Rescue had expected gender-based violence to rise amid pandemic and quarantines. Hence, the report had also recommended to prepare and build on existing services for the victims of gender-based violence. The report further emphasized on the need to strengthen online services to provide psychological support and legal aid services (19). According to Menendez et al. (20), often women do not have rights over their sexual choices. Consequently, they experience sexual violence and the risk of exposure to the virus through the male carrier. Okur (21) emphasized that sexual and gender-based violence increases during crisis situations due to breakdown in law. Thus, the victims often do not receive the adequate support, and the perpetrators get exempted from punishment. Also, according to the WHO global ethics unit (22), gender roles affect all aspects of an endemic including interpersonal violence. It also emphasized the need of various services to minimize the risk of violence when people are quarantined at home or in institutions. Hence, the present research shall focus on the gender-based violence, because despite being a global phenomenon, it is highly underreported due to stigma and social pressures. Moreover, there is a lack of studies focusing on the prevalence of gender-based violence during disasters. Consequently, those responding to disasters are often not aware of the possibility of surge in the cases of gender-based violence. Therefore, they often do not prepare to deal with, thereby making the situation worse. In fact, according to John et al. (23), these are the lessons never learnt. Therefore, we have a limited understanding toward how the victims of gender-based violence respond to the situation of the current pandemic. Hence, the present research reviews the linkages between gender violence and pandemic and also attempts to identify the potential policy responses to moderate the issue.

In the past, crises have been linked with a surge in cases of gender violence (24–27). A surge in intimate partner violence was observed during other disasters such as Earthquake in Haiti in 2007, Hurricane Katrina in 2005, and Eruption of Mount Saint Helens in the 1980s due to unemployment, family, and other stressors (28). Even during the South Asian Tsunami of 2004, a surge in gender-based violence was observed. Fisher (29) emphasized that in the aftermath of Tsunami, several incidents of violence against women and sexual assault were reported in Sri Lanka. According to researchers, pandemics cannot be considered an exception to this (9). Sikira and Urassa (30) reported an increase in wife battering in the face of the HIV pandemic due to suspicion of extramarital affairs. Recent outbreaks such as Ebola, Cholera, Zika, and Nipah have also led to an increase in the cases of domestic violence (31). During the Ebola virus outbreak, women and girls were especially vulnerable to violence because of the inability to escape their abuser. Moreover, the victims of violence were not recognized and were often left unattended (32). According to Yasmin (33), cases of rape, violence against women, and sexual assault also increased during the Ebola outbreak in West Africa.

There are a number of reasons for such increase in gender violence cases. Arthur and Clark (34) also identified economic dependence as a cause for domestic violence. During quarantine, as more women were in informal jobs and got laid off, this led to them experiencing a greater impact as they became economically dependent on their male counterparts. According to Alon et al. (35), lesser women than men are in telecommutable jobs, thus making it difficult for them to adapt to the changing conditions. This increased economic dependence not only increases their risk of gender-based violence but also makes it difficult to leave their perpetrators. Pandemics like influenza, swine flu, and SARS have been found to result in psychological issues such as anxiety, substance abuse, PTSD, and sleep disturbances that often tend to continue even after the pandemic (36, 37). According to a research by Zhang et al. (38), increased prevalence of depressive symptoms could be observed among COVID 19 patients. A significant rise in anxiety levels of the COVID-19 patients as well as the general public was reported by the findings of the study. In return, these mental health issues and related factors such as alcoholism tend to lead to a rise in gender-based violence (39–42). Several researchers have reported that the sales of alcohol have skyrocketed during pandemic [e.g., (43, 44)]. Polakovic (43) reported a rise of 55% in the consumption of alcoholic beverages in the United States. Evidence also suggests that increase in male migration reduces gender violence due to reduced exposure to the potential perpetrators (45). When under quarantine, women individuals are in close proximity to the male members with limited to no freedom to go out, thus leading to an increase in gender violence at home. Pandemics also increase economic vulnerabilities because of the rise in unemployment, or, in the risk of unemployment. Several studies link economic insecurities to increased gender-based violence. Economic insecurity has been found to be linked to adopting poor coping strategies that are inclusive of substance abuse (46–48). These, in turn, have been found to be associated with various forms of gender-based violence (49). However, interesting gender differences can be observed in this context. Bhalotra et al. (50) reported that increase in male unemployment was associated with increase in interpersonal violence against women where an increase in women unemployment was associated with a decrease in violence against them. According to Schneider et al. (51), such an outcome could be because of male backlash resulting from feelings of emasculation and inadequacy at not being able to serve the role of a breadwinner of the family. According to Bradbury-Jones and Isham (52), it could also be because of the distorted power dynamics at home resulting in abuse and gender violence that escapes the scrutiny of anyone from outside. The problem of gender-based violence during the pandemic further worsens because the police are unable to tackle the issue of gender-based violence. According to a report, gender-based violence in Liberia could have also increased because the police were overwhelmed and unable to defend the victims (53). Richards (54) reported that economic strain, substance abuse, and isolation all tend to increase the risk of domestic violence. Based on the above literature review, it is evident that understanding of gender violence is a key priority in order to achieve gender equality globally.

Past researches have established a strong link between different forms of gender-based violence and psychological issues. Thus, it is all the more important to tackle the issue of rising gender-based violence in the face of COVID-19. It has been reported that women who experience one form of gender-based violence are more likely to experience other forms of gender violence (55). According to Campbell (56), intimate partner violence is associated with PTSD, depression, chronic pain, sexually transmitted diseases, etc. Woods (57) reported that PTSD symptoms could be observed in both abused and post-abused women. Jackson et al. (58) established a link between traumatic brain injury and woman battering. They reported that the frequency of being hit in the head was significantly correlated with severe cognitive symptoms. Walker (59) reported that victims of domestic violence experience a sequelae of psychological symptoms that include anxiety, depression, avoidance, reexperiencing of traumatic events, and hyper-arousal.

## COVID-19 and Gender Violence

COVID-19 seems to be similar to the pandemics in the past since this too has resulted in an increase in cases of domestic violence. According to Bradbury-Jones and Isham (52), the lockdown imposed to deal with COVID-19 has granted greater freedom to abusers. Several media reports indicate a surge in cases of domestic violence in various countries. According to Kagi (60), though a drop was observed in the overall crime rates in Australia, the domestic abuse rates increased by 5%. Some charities in Australia also raised concerns about COVID-19 misinformation being used by the offenders to further control and abuse the victims of domestic violence (61). Allen-Ebrahimian (62) reported that China witnessed a three-fold increase in the cases of domestic violence after imposing quarantine. Different states in the United States also reported an increase of about 21–35% in domestic violence (63). Even the UK has been facing concerns due to rising family violence. There has also been an apparent increase in the number of domestic homicides (64). The Refuge website recorded an increase of 150% in the calls about domestic abuse (65). An article in The Indian Express draws attention to the fact that a vast majority of people in Mumbai do not have household water connections. With rising summer temperatures, people spending more time at homes during lockdowns, and emphasis on handwashing, there comes the need for household water. Consequently, many women are turning to underground water market operating under the cloak of darkness. Moreover, women have been spending more time queuing up for water and often approach the market in the wee hours of mornings where they often face verbal and sexual harassment (66). Despite this increase in incidents of gender-based violence, Jagori, a Delhi-based NGO, has witnessed a drop in calls on its helpline numbers by 50%. This could be because of the fear of getting discovered by their offenders at home according to Jaya Velankar, Director Jagori (67). According to Bradbury-Jones and Isham (52), the lockdown imposed to deal with COVID-19 has granted greater freedom to abusers. It has become easier for the abusers to enforce control tactics by limiting the access of the victims to phones, internet, and other people. van Gelder et al. (68) also emphasized that the lockdown limits familiar support options. In an article published by BU today (69), Rothman who is a professor of Community Health Sciences raised concerns about declaring sale of guns to be essential services in some states of the United States. This increases the likelihood of fatal interpersonal violence. Fielding (70) pointed out that the victims of abuse may even be scared to visit a hospital for treatment of their injuries due to the fear of contracting the COVID-19 disease.

## Tackling Gender-Based Violence During COVID-19

The first step to tackle the issue of rising gender violence in the times of pandemic is the acknowledgment of the issue, which has been ignored during the pandemics in the past (71). Campbell (28) emphasizes that expanding community partnerships and spreading awareness about the importance of reporting incidents of abuse are crucial to reducing the number of such cases. According to Bradbury and Isham (52), one way to deal with the issue of domestic violence is by constantly asking if people feel safe at home. However, it is very crucial that the people asking these questions have the time and emotional resources. It is often possible that the victims may communicate in subtle and indirect ways, which can be easily missed. They also emphasize the importance of online and telephonic services for those seeking therapeutic interventions, counseling, or any other kind of support. Gerster (72) emphasizes that neighbors of families with violence can also help to reduce domestic violence by initiating conversation with them. Researchers also emphasize the need to train healthcare workers to recognize the signs of violence to tackle the issue of gender-based violence (73, 74). Van Gelder et al. (68) emphasize the role of the media to raise awareness about the issue of gender violence during pandemic as well as about the practices that can replace the conventional in-person support. These may include offering supportive statements, promoting safety guidelines via advertisements, bystander approaches, and accessing help on behalf of the victim after obtaining consent. They also call for increase in service availability and funding for protection needs and shelters during quarantine. Hatchimonji et al. (71) called for coupling physical distancing with social support to ensure that it does not exacerbate gender violence. There is also a strong need to strengthen the helpline services which victims of gender violence can utilize without alerting their offenders. Antonio Guteres, the United Nations Secretary General, also emphasized the need for the countries to prioritize support by setting up emergency warning systems for individuals facing family violence (75). Mazza et al. (76) have emphasized on the need of a trained multidisciplinary staff including psychologists, psychiatrists, and social and legal services to prevent acts of domestic violence and ensure accurate assessment of various domains of the abuse.

Some countries have in fact tried to adapt to the situation of quarantine resulting from COVID-19 by implementing several practices to reduce gender-based violence. For instance, France has set up warning systems at groceries and pharmacies to enable victims of gender and family violence to alert the authorities (77). They may also alert the staff about the required help by using code words that have been introduced. Domestic Violence Resource Center Australia has also issued specific guidance for family and friends to support those in family violence situations (78). UNFPA (United Nations Population Fund) and UN Women have published guidelines that can be utilized by various governments to include gender considerations into their responses (15, 79). National Domestic Violence Hotline, USA, has also been offering service via online texting chat so that victims of domestic violence can seek help (80). In Beijing, a judicial court has been using cloud-based platforms and online court hearings to deal with cases of gender-based violence in the times of pandemic (81). Nair and Banerjee (82) emphasized the need for the combined efforts of health professionals with print and digital media to avoid misinformation and educate people about abuse prevention.

In a conversation with staff of AALI (Association for Advocacy and Legal Initiative, Lucknow, India), it was revealed that the actions being taken by the authorities in India are insufficient to deal with the issue of gender violence during COVID-19. NGOs have requested to publicize the phone numbers of the protection officers by sticking them outside their offices to make them more accessible to the victims. The AALI staff member also expressed concern over a lack of sense of urgency when dealing with domestic violence cases under lockdown. The effectiveness of the helplines is reduced if it is not followed by necessary action and is merely recorded as data. The National Commission of Women (NCW), India and NGOs such as Jagori have compiled information pertaining to the One Stop Centers, protection officers, and other support services on their websites. Aman: global Voices for Peace in the Home, which is a network of over 146 organizations and individuals working on the issue of violence against women across 18 states in India, has written a letter to the National Commission of Women, India with collective recommendations to respond to the situation of women facing violence under lockdown. The recommendations include making the helpline numbers such as 181 and 1,091 functional; publicizing the support services and resources available; utilizing Nirbhaya funds (Nirbhaya Fund is a corpus fund of Indian rupee 10 billion created by the Government of India to support the activities and initiatives of the government and NGOs working towards protecting the dignity and ensuring safety of women in India.) to increase the availability of resources available to NGOs offering legal aid, counseling, and shelter to women facing violence; developing special protocols to provide support to trans women, disabled women, and migrant women who are even more marginalized and have negligible access to support; and forming a panel of lawyers offering legal information to women over phone, among others. The Aman network has also recommended to build a temporary shelter in the Kashmir Valley, as there are no shelter homes built under the Protection of Women under the Domestic Violence Act, 2005.

The outcome of gender-based violence is long lasting for its victims, and rampant for the responses that are often inadequate. Hence, it is crucial to maintain a sense of urgency in cases of gender-based violence even during crisis situations. Based on the above literature review, it can be maintained that there is a need for a holistic response model to deal with the issue of gender-based violence during current and possible future pandemics. Health professionals, media, and community efforts must be combined to effectively deal with the issue of gender-based violence. Moreover, continuous and rigorous efforts are required to put an end to the stigma associated with gender-based violence.

## Conclusion

The spread of the novel Coronavirus has created a myriad of problems for the people to grapple with. In the absence of a vaccine and effective treatment for this virus, the governments are forced to impose quarantines to reduce the spread of the disease. However, this has resulted in a paradox of social distancing, which includes issues such as economic instability, mental health problems, and isolation. Although there have been researches exploring the impact of COVID-19, there is a lack of rigorous literature highlighting these issues from the perspective of gender. This also involves the issue of rising gender violence during the pandemic. COVID-19 has not only led to an increase in the cases of gender-based violence but has disconnected them from their support networks. To reduce the prevalence of the issue, it is crucial to acknowledge the extent of gender-based violence, reimagine government policies, and support networks to make it easier for the victims to access them and, lastly, create awareness about the issue as well as the resources available to tackle it.

## Author Contributions

SM and TS contributed to the conception, structure of the paper, contributed to analysis, and interpretation of available literature. SM contributed to the development initial draft. TS reviewed and critiqued the output for important intellectual content. All authors contributed to the article and approved the submitted version.

## Conflict of Interest

The authors declare that the research was conducted in the absence of any commercial or financial relationships that could be construed as a potential conflict of interest.
